# Test–Retest Reliability and Validity of a Sums-of-Gaussians-Based Markerless Motion Capture System for Human Lower-Limb Gait Kinematics

**DOI:** 10.3390/bioengineering13030271

**Published:** 2026-02-26

**Authors:** Yifei Shou, Chuang Gao, Chenbin Xi, Junqi Jia, Jiaojiao Lü, Yufei Fang, Chengte Lin, Zhiqiang Liang

**Affiliations:** 1 Faculty of Sports Science, Ningbo University, Ningbo 315211, China; 236000323@nbu.edu.cn (Y.S.); nbugaochuang@outlook.com (C.G.); 18969074840@163.com (C.X.); nbujiajunqi@outlook.com (J.J.); 2 Key Laboratory of Exercise and Health Sciences of Ministry of Education, Shanghai University of Sport, Shanghai 200438, China; lvjiaojiao@sus.edu.cn; 3 Department of Rehabilitation Medicine, Ningbo No. 2 Hospital, Ningbo 315010, China; fyf369@126.com; 4 State Key Laboratory of Advanced Marine Materials, Ningbo Institute of Materials Technology and Engineering (NIMTE), Chinese Academy of Sciences, Ningbo 315201, China; linzhengde@nimte.ac.cn

**Keywords:** markerless motion capture, sums-of-Gaussians body model, gait analysis, joint kinematics, reliability, concurrent validity, walking, slow running

## Abstract

Background and aim: Traditional marker-based optical motion capture systems are costly, time-consuming to operate, and constrained by laboratory environments, limiting their broader adoption in clinical practice and naturalistic settings. Markerless motion capture based on a sums-of-Gaussians (SoG) body model is a potential alternative; however, its metrological properties for kinematic assessment during walking and slow running remain insufficiently validated. Using a conventional marker-based Vicon system as the reference, this study evaluated the reliability and concurrent validity of an SoG-based markerless system (MocapGS) for bilateral lower-limb joint range of motion (ROM) during gait. Methods: Thirty-six healthy adults completed self-selected-pace speed walking and slow running tasks while both systems synchronously acquired bilateral lower-limb kinematics. The intraclass correlation coefficient (ICC), standard error of measurement (SEM), SEM percentage (SEM%), minimal detectable change (MDC), MDC percentage (MDC%), and root mean square error (RMSE) were used to assess reliability. Concurrent validity was evaluated using the Pearson correlation coefficient, paired-sample *t*-tests, and the concordance correlation coefficient (CCC) to compare the ROM. Results: Vicon showed moderate-to-high reliability for ROM in most joints across both tasks. By contrast, the MocapGS achieved acceptable ICC values mainly for the sagittal-plane ROM at the hip and knee. The CCC analysis showed no significant agreement between the two systems. Bland–Altman plots showed systematic biases with spatially heterogeneous random errors. During walking, MocapGS systematically overestimated ROM relative to Vicon at several joint axes; the widest limits of agreement (LOA) occurred at the left knee X-axis and right hip Z-axis. During running, overestimation was consistent across all bilateral joints at the X-axis and the right hip at the Y-axis, while the widest LOA were found at the bilateral hip X-axes. These specific discrepancies highlighted the joint–axis combinations with the greatest measurement variance. In walking, the test–retest reliability of the knee flexion–extension ROM measured by the MocapGS approached that of Vicon; however, the SEM% and MDC% were generally larger for MocapGS than for Vicon. The RMSE exceeded 5 degrees for ROM in most joint planes, especially in the frontal and transverse planes and at distal joints; errors increased further during slow running. Conclusions: MocapGS may be used for coarse monitoring of large-magnitude changes in sagittal-plane kinematics during gait; however, it is currently unlikely to replace Vicon for clinical decision-making or detecting subtle gait changes, and its outputs should be interpreted with caution, particularly for ankle kinematics and non-sagittal-plane motion.

## 1. Introduction

Precise quantification of human gait is a central topic in biomechanics, rehabilitation medicine, and motor control, and it is essential for assessing lower-limb functional status, identifying movement disorders, and optimizing training prescriptions [1,2,3]. High-quality three-dimensional gait kinematic acquisition for humans depends substantially on the performance of motion capture systems and their applicability across different settings [2,3]. Marker-based optical motion capture systems have well-recognized advantages in measurement accuracy, temporal resolution, and test–retest reliability under controlled laboratory conditions and are still widely regarded as one of the gold standards for human movement analysis [3,4,5]. Nevertheless, their susceptibility to soft-tissue artifacts, strict requirements for operator expertise, clothing and environment constraints, and relatively complex testing procedures have limited their wider use in naturalistic environments and large-sample studies [4,6,7].

With advances in computer vision, machine learning, and multi-view 3D reconstruction, markerless motion capture has increasingly become an important complement to marker-based systems [2,3,4,5,8,9]. Markerless systems typically integrate multi-camera video/depth information with human body models and pose estimation algorithms to automatically identify skeletal pose and reconstruct kinematics [8,9]. Compared with marker-based systems, markerless approaches offer the clear advantages of simplified workflows, reduced dependence on clothing and lab conditions, and better preservation of natural movement, making them particularly suitable for multi-speed, multi-task movement analyses and long-term monitoring [2,8,9]. However, because markerless systems differ substantially in their modeling strategies, algorithm robustness, and quantitative accuracy, systematic comparative studies are needed to establish whether their kinematic outputs are sufficiently consistent with gold-standard systems [3,5,8,9].

The sums-of-Gaussians body model (SoG) is a recently developed representative modeling framework for markerless motion capture [10,11,12,13]. This approach parameterizes body segment shape and appearance using a set of Gaussian distributions and performs continuous pose tracking via optimization under multi-camera image constraints [10,11,12]. Compared with approaches that rely heavily on large-scale training data or complex silhouette extraction, SoG-based modeling may require fewer prior data and achieve 3D pose estimation at relatively low computational cost, providing a favorable balance between reconstruction accuracy and real-time performance [10,11,12]. Existing studies suggest that SoG models can achieve a degree of concurrent validity and reliability in joint kinematics reconstruction in algorithmic and simulation contexts [10,11,12,13]. However, systematic quantitative validation of real human periodic movements, particularly walking and running across different speeds, remains limited, which hinders standardized clinical gait analysis and broader methodological adoption for performance assessment.

Therefore, this study used a conventional marker-based optical motion capture system as a reference under unified experimental conditions and compared SoG-based markerless motion capture with marker-based motion capture during two typical periodic lower-limb tasks (walking and running). We aimed to (i) compare bilateral lower-limb joint kinematics outputs and (ii) quantify agreement between the systems across tasks, thereby systematically evaluating the feasibility and applicability of SoG-based markerless motion capture for walking and running gait analysis. This work is intended to provide methodological evidence and empirical support for the standardized use of SoG-based markerless motion capture in clinical gait evaluation, rehabilitation monitoring, and biomechanical research. In addition to supporting clinical and laboratory gait assessment, establishing the metrological boundaries of markerless kinematics is directly relevant to field-ready applications such as footwear biomechanics evaluation and sport injury prevention, where rapid, scalable gait screening is often needed.

## 2. Materials and Methods

### 2.1. Participants

Thirty-six healthy adult university students (height: 171.20 ± 8.04 cm; mass: 59.90 ± 7.28 kg) were recruited. Inclusion criteria were as follows: (1) age ≥ 18 years; (2) right leg dominance; (3) regular physical activity (≥3 sessions/week; ≥30 min/session); and (4) no lower-limb injury within the past six months and no other diseases that could affect exercise capacity. Exclusion criteria were as follows: (1) minors; (2) sedentary individuals; and (3) individuals with habitual late-night routines or other high-risk characteristics for exercise, which aimed to minimize the potential confounding effects of circadian rhythm disruption on gait. The study was approved by the Ethics Committee of Ningbo University (Approval No. TY2025010).

### 2.2. Experimental Procedure

A randomized crossover experimental design was used. All participants visited the laboratory twice. The first visit was used to collect basic information and familiarize participants with the experimental procedure and testing tasks. During the second visit, participants performed walking and slow running tasks in a randomized order. All walking and slow running trials were performed on a level laboratory floor. For each task, participants completed two sets of three trials at a self-selected speed. During all trials, bilateral lower-limb joint kinematics were synchronously recorded using the MocapGS 3D markerless system (China) based on SoG and the Vicon 3D infrared optical system (UK). ROM over the gait cycle was calculated for subsequent statistical analyses.

### 2.3. Marker-Based Infrared Optical Motion Capture System

An eight-camera Vicon motion capture system was used to collect 3D kinematics of the hip, knee, and ankle joints bilaterally during walking and slow running. The built-in Plug-in Gait model was adopted, and the sampling frequency was 200 Hz. Before data acquisition, 16 reflective markers were attached to anatomical landmarks according to Plug-in Gait requirements: left anterior superior iliac spine, right anterior superior iliac spine, left posterior superior iliac spine, right posterior superior iliac spine, left thigh, right thigh, left knee, right knee, left tibia, right tibia, left ankle, right ankle, left toe, right toe, left heel, and right heel.

### 2.4. Markerless Motion Capture System

A four-camera MocapGS (China) markerless motion capture system was used to capture 3D kinematics of the hip, knee, and ankle joints bilaterally during walking and running at 50 Hz. This system employs the SoG algorithm to automatically track the participant and build a skeletal model; its software provides real-time visual feedback on the skeletal model overlay. During gait measurement, the four cameras were positioned at the four corners of the capture volume on vertical mounts, with their lenses angled inward at 65° relative to the mount. A dynamic calibration procedure using a predefined wand was performed before each testing session to define the capture space (5 m in length and 3 m in width) and synchronize the cameras. Consistent with the Vicon protocol, participants wore tight-fitting, single-color athletic clothing, and the MocapGS system recorded the bilateral lower-limb kinematics during walking and slow running without requiring participants to wear any markers. After data collection, kinematics were computed via automatic matching to a static skeletal model and exported in C3D format.

### 2.5. Ground Reaction Force Measurement

Vertical ground reaction force (GRF) data were collected using two force plates (AMTI, Watertown, MA, USA) embedded in the walkway. The force plates were sampled at 1000 Hz. The raw GRF signals were filtered using a fourth-order zero-lag Butterworth low-pass filter with a cutoff frequency of 50 Hz. Gait events (initial contact and toe-off) were identified automatically for each step by the force plate. Initial contact was defined as the instant the vertical GRF exceeded 20 N, and toe-off was defined as the instant it fell below 20 N. The force plate system was synchronized with both the Vicon and MocapGS motion capture systems via an external analog trigger signal, ensuring the temporal alignment of all data streams for subsequent analysis.

### 2.6. Data Processing and Statistical Analysis

#### 2.6.1. Data Processing

After preliminary processing using the proprietary software of each system, 3D joint kinematics were imported into Visual3D (v3.21.0; C-Motion, Inc., Germantown, MD, USA). A participant-specific lower-limb skeletal model was built based on the static trial. Data of the lower-limb joint kinematics were filtered using a fourth-order zero-lag Butterworth low-pass filter with a cutoff frequency of 10 Hz. Gait events were automatically identified using vertical ground reaction force. Each walking and slow running bout was segmented into independent gait cycles to compute the 3D ROM for each joint.

#### 2.6.2. Statistical Analysis

All ROM data were reported as mean ± standard deviation (M ± SD). Normality was assessed using the Shapiro–Wilk test; nonparametric tests were applied if normality assumptions were violated.

Relative reliability was assessed using the ICCs for the ROM of the hip, knee, and ankle along the X-, Y-, and Z-axes bilaterally. ICC values were computed by comparing the first three trials versus the last three trials. The ICC was calculated using a two-way mixed-effects model for absolute agreement, where each measurement was based on the mean of three trials (k = 3). Reliability thresholds were ICC > 0.75 (high), 0.40 < ICC ≤ −0.75 (moderate), and ICC < 0.40 (low).

Absolute reliability was quantified using the SEM, SEM%, MDC, and MDC%. The SEM was also derived from the ICC model to assess measurement precision. SEM% and MDC% were calculated as SEM% = (SEM/Mean ROM) × 100% and MDC% = (MDC/Mean ROM) × 100%, respectively. The mean ROM was the average from the corresponding system for each joint. Descriptive statistics were used to compare the above parameters to evaluate the measurement precision and sensitivity between systems.

Concurrent validity was evaluated using Pearson’s correlations and paired-sample *t*-tests. The RMSE was calculated to quantify differences between the measurements from the two systems. The CCC was calculated to assess the agreement between the two measurement systems. Bland–Altman analysis was performed to quantify the mean systematic bias (Vicon minus MocapGS) and the 95% LOA, with the SD of differences and LOA width used to assess random error magnitude across joints, axes, and lower-limb sides. Correlation strength was interpreted as 0.8 < r ≤ 1.0 (very strong), 0.6 < r ≤ 0.8 (strong), 0.4 < r ≤ 0.6 (moderate), and r ≤ 0.4 (weak). The CCC threshold was consistent with the ICC threshold. All analyses were conducted in SPSS 21.0 (IBM Inc., Armonk, NY, USA), with α = 0.05.

## 3. Results

### 3.1. Relative Reliability of Lower-Limb Joint Kinematics Measured by the Two Systems During Walking

**Figure 1:**
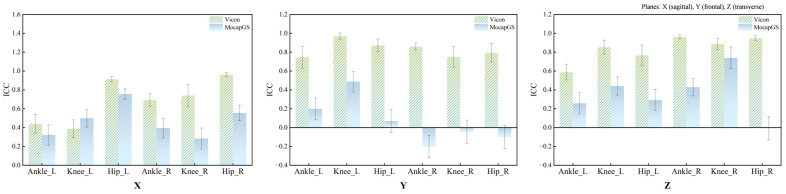
ICC values for ROM measured by the Vicon and MocapGS systems during walking. X is the sagittal plane; Y is the frontal plane; Z is the transverse plane; Ankle_L is the left ankle joint; Ankle_R is the right ankle joint; Knee_L is the left knee joint; Knee_R is the right knee joint; Hip_L is the left hip joint; and Hip_R is the right hip joint.

Figure 1 and Table 1 summarize the ICCs for the ROM of the bilateral hip, knee, and ankle joints along the X-, Y-, and Z-axes during walking. Vicon produced statistically significant reliability for the ROM of the three lower-limb joints (*p* < 0.05); except for the comparatively lower ICCs for the left ankle and left knee along the X-axis, most joint axes achieved at least moderate reliability. In contrast, the MocapGS showed statistically significant reliability (*p* < 0.05) only for selected joint axes. Specifically, the ICC for the left hip ROM along the X-axis was high, whereas the left knee ROM along the X-, Y-, and Z-axes showed moderate ICCs, with the knee X-axis ICC exceeding that of Vicon. On the right side, the knee ROM along the Z-axis approached a high ICC, while the hip ROM along the X-axis and the ankle ROM along the Z-axis showed moderate ICCs.

### 3.2. Absolute Reliability of Lower-Limb Joint Kinematics Measured by the Two Systems During Walking

**Figure 2:**
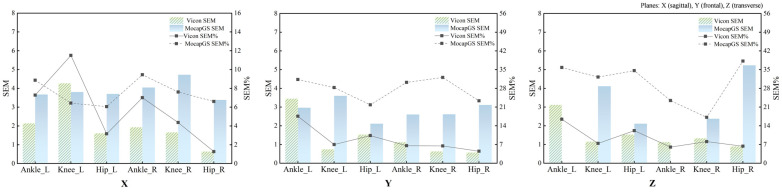
The SEM and SEM% for ROM measured by the Vicon and MocapGS systems during walking. X is the sagittal plane; Y is the frontal plane; Z is the transverse plane; Ankle_L is the left ankle joint; Ankle_R is the right ankle joint; Knee_L is the left knee joint; Knee_R is the right knee joint; Hip_L is the left hip joint; and Hip_R is the right hip joint.

**Figure 3:**
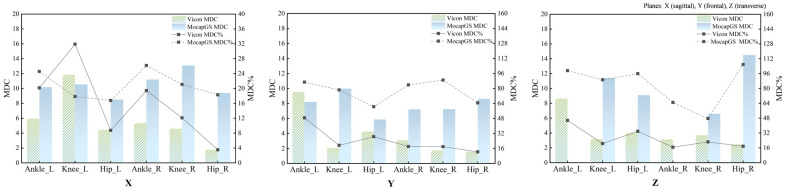
The MDC and MDC% for ROM measured by the Vicon and MocapGS systems during walking. X is the sagittal plane; Y is the frontal plane; Z is the transverse plane; Ankle_L is the left ankle joint; Ankle_R is the right ankle joint; Knee_L is the left knee joint; Knee_R is the right knee joint; Hip_L is the left hip joint; and Hip_R is the right hip joint.

Absolute reliability of the bilateral hip, knee, and ankle ROM during walking was quantified using the SEM, SEM%, MDC, and MDC%. Figure 2 and Figure 3 and Table 2 and Table 3 report the SEM, SEM%, MDC, and MDC% for ROM of the bilateral hip, knee, and ankle joints during walking.

#### 3.2.1. Comparison of Measurement Precision During Walking

Descriptive comparisons of measurement precision (Figure 2 and Table 2) indicated that, compared to Vicon, MocapGS produced a smaller SEM for the left ankle ROM along the Y- and Z-axes, the left knee ROM along the X-axis, and the right ankle ROM along the Z-axis. When normalized to ROM, MocapGS showed a lower SEM% for the left knee X-axis than Vicon. For the remaining joint axes, neither the SEM nor SEM% from MocapGS surpassed those obtained with Vicon.

#### 3.2.2. Comparison of Measurement Sensitivity During Walking

Regarding measurement sensitivity (Figure 3 and Table 3), MocapGS produced a smaller MDC than Vicon for the left ankle ROM along the Y- and Z-axes and for the left knee ROM along the X-axis; the MDC% for the left knee X-axis ROM was lower for MocapGS. For the right limb, MocapGS produced a smaller MDC for the ankle Z-axis ROM than Vicon, whereas there was no MDC% advantage for MocapGS over Vicon.

### 3.3. Concurrent Validity of Lower-Limb Joint Kinematics Measured by the Two Systems During Walking

#### 3.3.1. Correlation Analysis of ROM Between Systems During Walking

**Figure 4:**
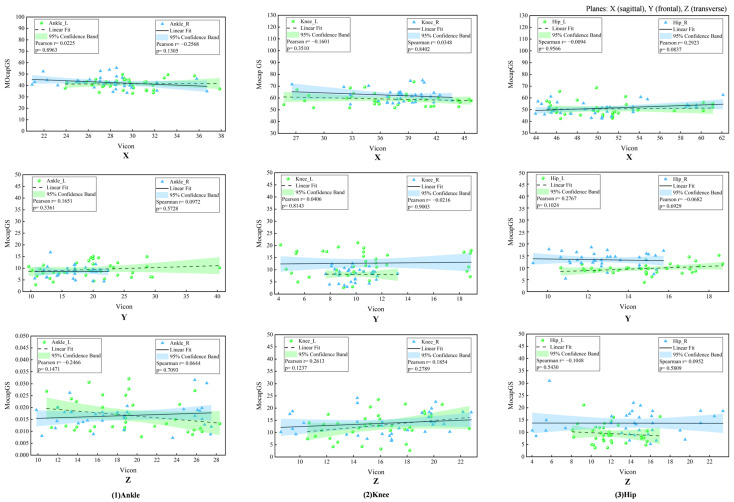
Correlation analyses of ROM measured by the Vicon and MocapGS systems during walking. X is the sagittal plane; Y is the frontal plane; Z is the transverse plane; Ankle_L is the left ankle joint; Ankle_R is the right ankle joint; Knee_L is the left knee joint; Knee_R is the right knee joint; Hip_L is the left hip joint; and Hip_R is the right hip joint.

The Pearson correlation analysis did not show any significant correlations in the joint ROM between MocapGS and Vicon during walking (all *p* > 0.05, Figure 4).

#### 3.3.2. Comparisons of ROM Between Systems During Walking

**Figure 5:**
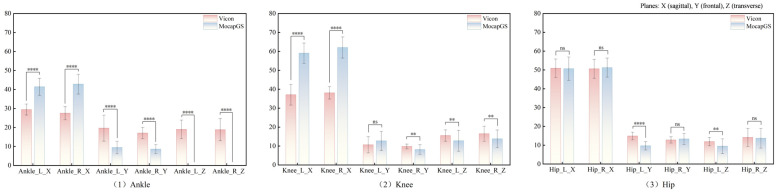
Comparisons of the ROM measured by the Vicon and MocapGS systems during walking. Ankle_L_X is the left ankle joint in the sagittal plane; Ankle_R_X is the right ankle joint in the sagittal plane; Ankle_L_Y is the left ankle joint in the frontal plane; Ankle_R_Y is the right ankle joint in the frontal plane; Ankle_L_Z is the left ankle joint in the transverse plane; Ankle_R_Z is the right ankle joint in the transverse plane; Knee_L_X is the left knee joint in the sagittal plane; Knee_R_X is the right knee joint in the sagittal plane; Knee_L_Y is the left knee joint in the frontal plane; Knee_R_Y is the right knee joint in the frontal plane; Knee_L_Z is the left knee joint in the transverse plane; Knee_R_Z is the right knee joint in the transverse plane; Hip_L_X is the left hip joint in the sagittal plane; Hip_R_X is the right knee joint in the sagittal plane; Hip_L_Y is the left hip joint in the frontal plane; Hip_R_Y is the right hip joint in the frontal plane; Hip_L_Z is the left hip joint in the transverse plane; and Hip_R_Z is the right hip joint in the transverse plane. Significance levels are coded as * for *p* < 0.05; ** for *p* < 0.01; *** for *p* < 0.001; **** for *p* < 0.0001; ns denotes *p* > 0.05.

Paired-sample *t*-tests (Figure 5 and Table 4) showed significant between-system differences for the bilateral ankle ROM along the X-, Y-, and Z-axes; the left knee ROM along the X- and Z-axes; the right knee ROM along the X-, Y-, and Z-axes; and the left hip ROM along the Y- and Z-axes (*p* < 0.05). MocapGS showed larger ankle ROM along the X-axis bilaterally and greater knee ROM along the X-axis bilaterally than Vicon. MocapGS showed smaller ankle ROM along the Y- and Z-axes, smaller right knee ROM along the Y-axis, smaller bilateral knee ROM along the Z-axis, and smaller left hip ROM along the Y- and Z-axes than Vicon.

**Figure 6:**
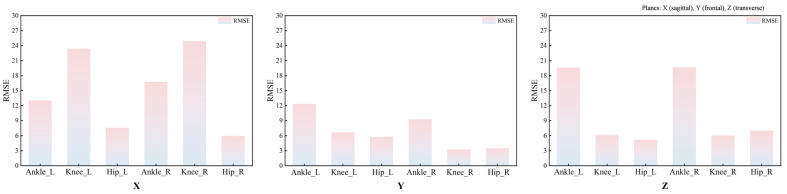
RMSEs between the ROM measured by the Vicon and MocapGS systems during walking. X is the sagittal plane; Y is the frontal plane; Z is the transverse plane; Ankle_L is the left ankle joint; Ankle_R is the right ankle joint; Knee_L is the left knee joint; Knee_R is the right knee joint; Hip_L is the left hip joint; and Hip_R is the right hip joint.

RMSE analyses further indicated systematic between-system discrepancies in ROM during walking (Figure 6 and Table 4). The mean RMSE across the three joints of the left lower limb was 11.09°, with joint-specific mean RMSEs of 6.19° at the hip, 12.08° at the knee, and 15.01° at the ankle. For the right lower limb, the mean RMSE across the three joints was 10.63°, with joint-specific mean RMSEs of 5.49° at the hip, 11.14° at the knee, and 15.24° at the ankle. The smallest discrepancies were observed in the Y-axis ROM at the knee and hip, both below 5°.

#### 3.3.3. Agreement Analysis of ROM Between Systems During Walking

**Figure 7:**
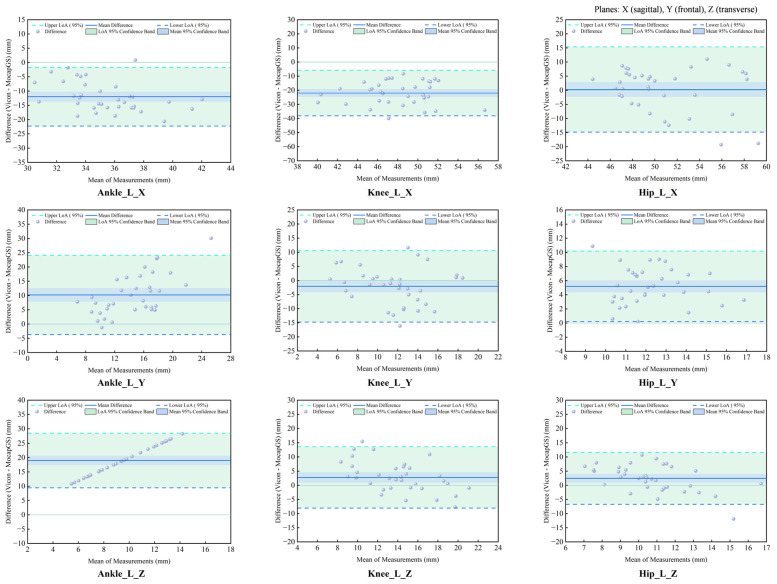
Bland–Altman plots of ROM agreement between the Vicon and MocapGS systems during walking for the left lower limb. Ankle_L_X is the left ankle joint in the sagittal plane; Ankle_L_Y is the left ankle joint in the frontal plane; Ankle_L_Z is the left ankle joint in the transverse plane; Knee_L_X is the left knee joint in the sagittal plane; Knee_L_Y is the left knee joint in the frontal plane; Knee_L_Z is the left knee joint in the transverse plane; Hip_L_X is the left hip joint in sagittal plane; Hip_L_Y is the left hip joint in the frontal plane; Hip_L_Z is the left hip joint in the transverse plane.

**Figure 8:**
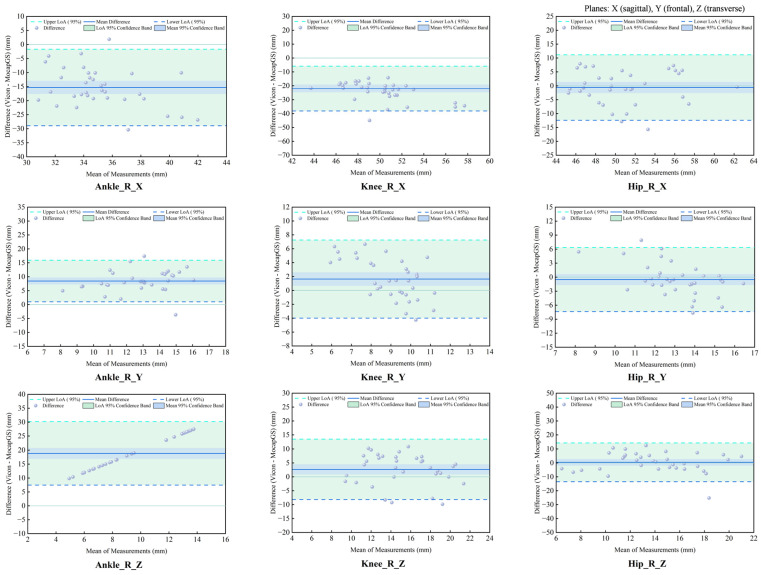
Bland–Altman plots of ROM agreement between the Vicon and MocapGS systems during walking for the right lower limb. Ankle_R_X is the right ankle joint in the sagittal plane; Ankle_R_Y is the right ankle joint in the frontal plane; Ankle_R_Z is the right ankle joint in the transverse plane; Knee_R_X is the right knee joint in the sagittal plane; Knee_R_Y is the right knee joint in the frontal plane; Knee_R_Z is the right knee joint in the transverse plane; Hip_R_X is the right hip joint in the sagittal plane; Hip_R_Y is the right hip joint in the frontal plane; Hip_R_Z is the right hip joint in the transverse plane.

The CCC and Bland–Altman analyses showed the patterns of measurement agreement between the MocapGS and Vicon systems across all lower-limb joints and planes (Figure 7 and Figure 8, Table 5). The CCC results indicated no significant consistency between the two systems (*p* > 0.05).

The Bland–Altman analysis found axis-dependent systematic bias and spatial heterogeneity in random errors between the Vicon and MocapGS for ROM. The axis-dependent systematic bias, as reported by the mean difference (MD), showed that MocapGS overestimated the left lower-limb ROM at the ankle X-axis and knee X- and Y-axes and underestimated ROM at the ankle Y- and Z-axes, knee Z-axis, and all hip axes, compared to Vicon. MocapGS overestimated the right lower-limb ROM at the ankle X-axis, knee X-axis, and hip X- and Y-axes and underestimated ROM at the remaining joint axes, compared to Vicon. The random errors, as represented by the SD and 95% LOA, showed spatial heterogeneity. The knee X- and Z-axes and the ankle Y-axis of the left lower limb showed larger random errors. For the right lower limb, the ankle X- and Y-axes and the hip Z-axis showed larger random errors.

### 3.4. Relative Reliability of Lower-Limb Joint Kinematics Measured by the Two Systems During Slow Running

**Figure 9:**
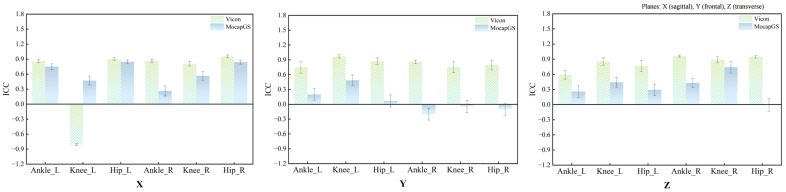
The ICCs for ROM measured by the Vicon and MocapGS systems during slow running. X is the sagittal plane; Y is the frontal plane; Z is the transverse plane; Ankle_L is the left ankle joint; Ankle_R is the right ankle joint; Knee_L is the left knee joint; Knee_R is the right knee joint; Hip_L is the left hip joint; and Hip_R is the right hip joint.

Table 6 summarizes the ICCs for the ROM of the bilateral hip, knee, and ankle joints along the X-, Y-, and Z-axes during slow running. The Vicon system produced 15 statistically significant ICCs (*p* < 0.05), including 8 for the left lower limb and 7 for the right lower limb, with ICCs overall falling within the moderate-to-high reliability range (Figure 9). By contrast, the MocapGS system produced seven statistically significant ICCs (*p* < 0.05), including four for the left lower limb and three for the right lower limb. On the left lower limb, the ankle and hip X-axis ROM showed high reliability, and the knee X-axis ROM and ankle Y-axis ROM showed moderate reliability. On the right lower limb, the hip X-axis ROM showed high reliability, and the knee X-axis ROM and ankle Y-axis ROM showed moderate reliability.

### 3.5. Absolute Reliability of Lower-Limb Joint Kinematics Measured by the Two Systems During Slow Running

#### 3.5.1. Measurement Precision Between Systems During Slow Running

**Figure 10:**
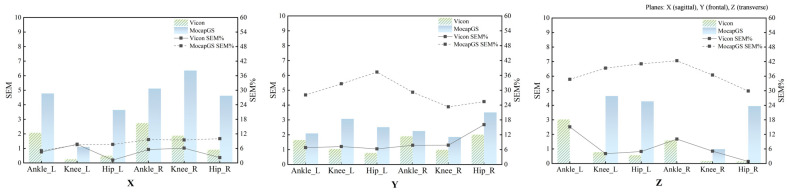
The SEM and SEM% for ROM measured by the Vicon and MocapGS systems during slow running. X is the sagittal plane; Y is the frontal plane; Z is the transverse plane; Ankle_L is the left ankle joint; Ankle_R is the right ankle joint; Knee_L is the left knee joint; Knee_R is the right knee joint; Hip_L is the left hip joint; and Hip_R is the right hip joint.

Figure 10 summarizes the SEM and SEM% measurements across the joints and planes during slow running. Descriptive comparisons of measurement precision during slow running (Figure 10 and Table 7) found that, compared to Vicon, MocapGS produced a smaller SEM for the knee ROM along the Z-axis bilaterally. However, the SEM% did not favor MocapGS for these variables, and an SEM% advantage was observed only for the left knee ROM along the X-axis. For the remaining joint axes, MocapGS did not show a lower SEM or SEM% than Vicon.

#### 3.5.2. Comparisons of Measurement Sensitivity Between Systems During Slow Runing

**Figure 11:**
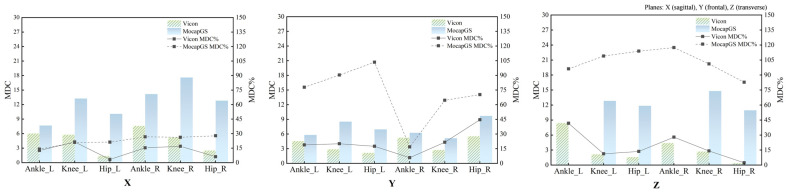
The MDC and MDC% for ROM measured by the Vicon and MocapGS systems during slow running. X is the sagittal plane; Y is the frontal plane; Z is the transverse plane; Ankle_L is the left ankle joint; Ankle_R is the right ankle joint; Knee_L is the left knee joint; Knee_R is the right knee joint; Hip_L is the left hip joint; and Hip_R is the right hip joint.

Figure 11 summarizes the MDC and MDC% across the joints and planes during slow running. Descriptive comparisons of measurement sensitivity (Figure 11 and Table 7) found that MocapGS produced a smaller MDC for the ankle ROM along the Z-axis bilaterally compared with Vicon. Nevertheless, MocapGS did not outperform Vicon in the MDC% for any lower-limb ROM.

### 3.6. Concurrent Validity of Lower-Limb Joint Kinematics Measured by the Two Systems During Slow Running

#### 3.6.1. Correlation Analysis of ROM Between Systems During Slow Runing

**Figure 12:**
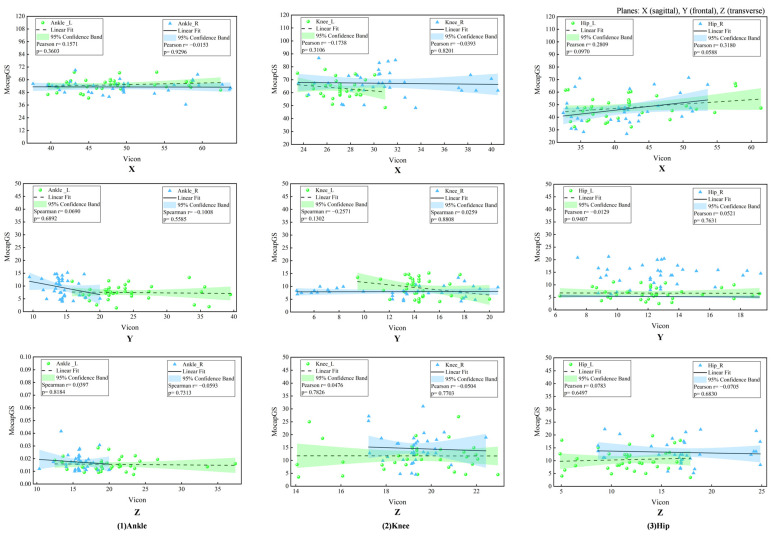
Correlation analyses of ROM measured by the Vicon and MocapGS systems during running. X is the sagittal plane; Y is the frontal plane; Z is the transverse plane; Ankle_L is the left ankle joint; Ankle_R is the right ankle joint; Knee_L is the left knee joint; Knee_R is the right knee joint; Hip_L is the left hip joint; and Hip_R is the right hip joint.

The Pearson correlation analysis showed no significant correlations between MocapGS- and Vicon-derived joint ROM during slow running (all *p* > 0.05; Figure 12).

#### 3.6.2. Comparisons of ROM Between Systems During Slow Runing

**Figure 13:**
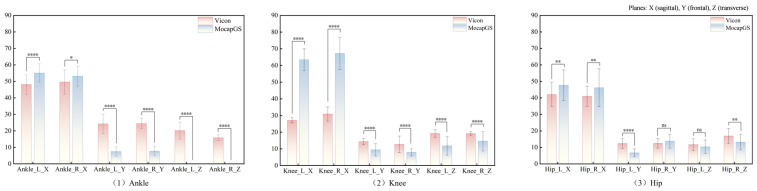
Comparisons of ROM measured by the Vicon and MocapGS systems during slow running. In the figure, Ankle_L_X is the left ankle joint in the sagittal plane; Ankle_R_X is the right ankle joint in the sagittal plane; Ankle_L_Y is the left ankle joint in the frontal plane; Ankle_R_Y is the right ankle joint in the frontal plane; Ankle_L_Z is the left ankle joint in the transverse plane; Ankle_R_Z is the right ankle joint in the transverse plane; Knee_L_X is the left knee joint in the sagittal plane; Knee_R_X is the right knee joint in the sagittal plane; Knee_L_Y is the left knee joint in the frontal plane; Knee_R_Y is the right knee joint in the frontal plane; Knee_L_Z is the left knee joint in the transverse plane; Knee_R_Z is the right knee joint in the transverse plane; Hip_L_X is the left hip joint in the sagittal plane; Hip_R_X is right knee joint in the sagittal plane; Hip_L_Y is the left hip joint in the frontal plane; Hip_R_Y is the right hip joint in the frontal plane; Hip_L_Z is the left hip joint in the transverse plane; and Hip_R_Z is the right hip joint in the transverse plane. Significance levels are coded as * for *p* < 0.05; ** for *p* < 0.01; *** for *p* < 0.001; **** for *p* < 0.0001; ns denotes *p* > 0.05.

Paired-sample *t*-tests comparing the joint ROM between the two systems during slow running (Figure 13 and Table 8) showed no significant between-system differences for the left hip ROM along the Z-axis or for the right hip ROM along the Y-axis (*p* > 0.05). In contrast, ROM about all remaining joint axes for both limbs differed significantly between systems (*p* < 0.05). Specifically, MocapGS reported significantly greater ankle and knee ROM along the X-axis bilaterally than Vicon, whereas the ankle and knee ROMs along the Y- and Z-axes were significantly smaller than those measured by Vicon (*p* < 0.05). For the hip, MocapGS-derived ROM along the X-axis was significantly greater than Vicon bilaterally, while the hip ROM along the Y-axis on the left and along the Z-axis on the right was significantly smaller than Vicon (*p* < 0.05).

**Figure 14:**
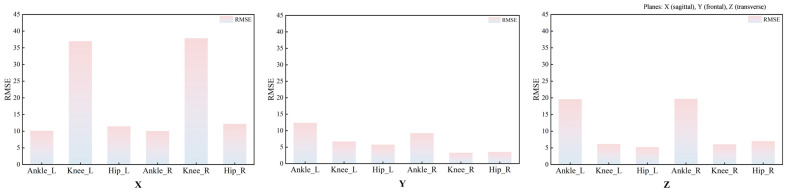
The RMSEs between ROM measured by the Vicon and MocapGS systems during slow running. X is the sagittal plane; Y is the frontal plane; Z is the transverse plane; Ankle_L is the left ankle joint; Ankle_R is the right ankle joint; Knee_L is the left knee joint; Knee_R is the right knee joint; Hip_L is the left hip joint; and Hip_R is the right hip joint.

RMSE analysis further quantified these discrepancies (Table 8 and Figure 14). The overall RMSE difference across the left lower-limb joint ROM reached 13.96°, with mean joint-specific RMSEs of 7.84° at the hip, 17.74° at the knee, and 16.29° at the ankle. For the right lower limb, the overall RMSE difference across the joint ROM reached 13.40°, with mean RMSEs of 8.31°at the hip, 17.48° at the knee, and 14.40°at the ankle.

#### 3.6.3. Agreement Analysis of ROM Between Systems During Slow Runing

**Figure 15:**
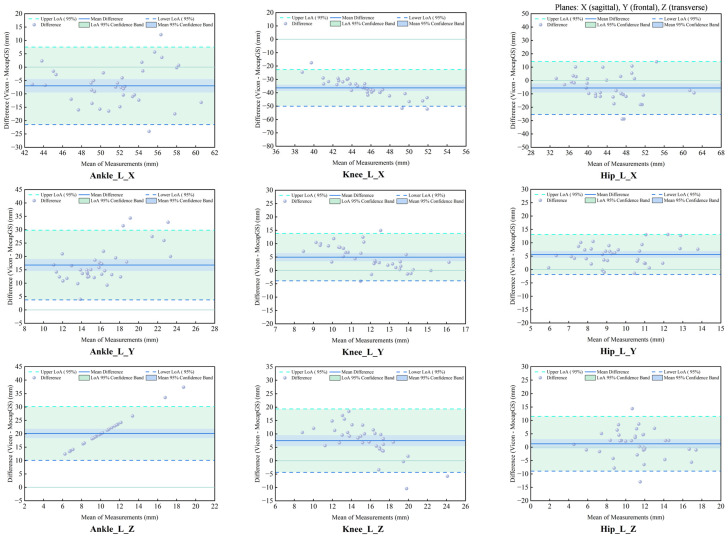
Bland–Altman plots of ROM agreement between the Vicon and MocapGS systems during slow running for the left lower limb. Ankle_L_X is the left ankle joint in the sagittal plane; Ankle_L_Y is the left ankle joint in the frontal plane; Ankle_L_Z is the left ankle joint in the transverse plane; Knee_L_X is the left knee joint in the sagittal plane; Knee_L_Y is the left knee joint in the frontal plane; Knee_L_Z is the left knee joint in the transverse plane; Hip_L_X is the left hip joint in the sagittal plane; Hip_L_Y is the left hip joint in the frontal plane; Hip_L_Z is the left hip joint in the transverse plane.

**Figure 16:**
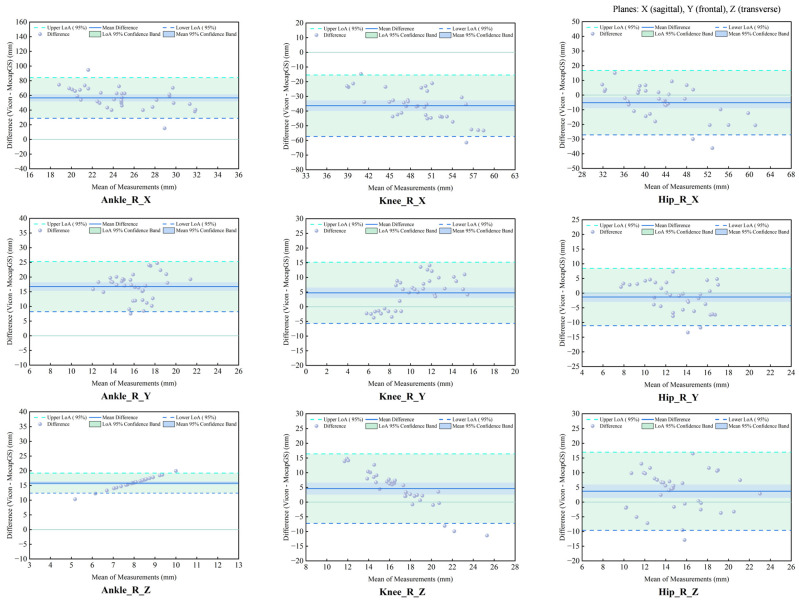
Bland–Altman plots of ROM agreement between the Vicon and MocapGS systems during slow running for the right lower limb. Ankle_R_X is the right ankle joint in the sagittal plane; Ankle_R_Y is the right ankle joint in the frontal plane; Ankle_R_Z is the right ankle joint in the transverse plane; Knee_R_X is the right knee joint in the sagittal plane; Knee_R_Y is the right knee joint in the frontal plane; Knee_R_Z is the right knee joint in the transverse plane; Hip_R_X is the right hip joint in the sagittal plane; Hip_R_Y is the right hip joint in the frontal plane; Hip_R_Z is the right hip joint in the transverse plane.

The agreement analysis of the slow-running ROM showed systematic measurement discrepancies between the MocapGS and Vicon systems across all joints (Figure 15 and Figure 16, Table 9). The CCC results indicated no significant consistency between the two systems (*p* > 0.05).

Bland–Altman analysis found axis-dependent systematic bias and spatial heterogeneity in random errors between the Vicon and MocapGS systems for measuring lower limb joint ROM. MocapGS overestimated the left lower-limb ROM at the ankle, knee, and hip X-axes and underestimated the left lower-limb ROM at all joint Y- and Z-axes, compared to Vicon. MocapGS overestimated the right lower-limb ROM at the ankle, knee, and hip X-axes and hip Y-axis and underestimated the right lower-limb ROM at all remaining joint axes, compared to Vicon.

The random errors, as represented by the SD and 95% LOA width, showed spatial heterogeneity. The hip X-axis, ankle Y-axis, and knee Z-axis of the left lower limb showed larger random errors. The hip X- and Z-axes and knee Y-axis of the right lower limb showed larger random errors.

## 4. Discussion

This study evaluated the feasibility of MocapGS, an SoG-based markerless motion capture system, for human gait analysis by comparing bilateral lower-limb joint ROM with a Vicon system during walking and slow running. The results indicated that MocapGS exhibited acceptable reliability primarily in the sagittal plane for hip and knee ROM. However, its concurrent validity and consistency remained significantly distinct from those of the Vicon system, especially in the frontal and transverse planes. These findings suggest that the current implementation of MocapGS does not achieve a level of measurement reliability and concurrent validity comparable to that of the Vicon system for human gait analysis. There is still much room for improvement in quantitative gait assessment.

### 4.1. Reliability and Concurrent Validity

The reliability performance observed for the Vicon is consistent with prior work showing that marker-based optical systems provide relatively stable joint ROM estimates under controlled conditions, particularly in the sagittal plane [3,14]. In contrast, the reduced reliability of MocapGS for frontal and transverse plane ROM aligns with the broader literature reporting that markerless systems are more sensitive to occlusion, pose ambiguity, and model-fitting instability for rotations that are comparatively small in amplitude and more susceptible to noise [3,9,15,16,17,18]. These limitations may be amplified during running because of higher segment velocities, larger inter-frame motion, and increased limb self-occlusion, which can degrade multi-view correspondence and tracking stability.

Beyond reliability, the concurrent validity analyses revealed a fundamental limitation in agreement between the systems. This finding is compounded by the lack of measurement consistency and the Bland–Altman analysis, which quantified substantial systematic biases [19,20,21,22]. These results indicate that the discrepancies captured by the elevated RMSE are not merely random but include consistent directional errors. Consequently, the MocapGS, in its current implementation, cannot be considered metrologically interchangeable with the marker-based Vicon system for quantitative gait assessment where precise multi-planar kinematics are required.

### 4.2. Joint-Specific Performance and Methodological Considerations

MocapGS exhibited only moderate-to-high ICCs for sagittal ROM at the hip and knee during walking and slow running, with knee sagittal ROM during walking approaching the reliability of Vicon. This limited agreement indicates that the SoG model merely captures gross flexion–extension patterns at proximal joints, rather than providing high-fidelity kinematic data. While this observation superficially aligns with reports that markerless methods perform adequately for high signal-to-noise movements [9,14,17], it also underscores a fundamental constraint: even under favorable conditions, MocapGS yields a larger SEM% and MDC% than Vicon, reflecting poor measurement precision and an inadequate sensitivity to detect clinically meaningful changes.

In contrast to the proximal joints, more pronounced limitations were observed at the ankle. MocapGS showed low reliability for ankle sagittal ROM during walking and for ankle frontal ROM during slow running. Moreover, no significant correlations between systems were found for the ankle frontal ROM in either task. These discrepancies are consistent with the known challenges of image-based tracking for distal segments, including motion blur, occlusion by the contralateral limb, and limited visual features. Small rotational ROMs at the ankle in the frontal and transverse planes are particularly susceptible to being obscured by reconstruction error [9,16,17,18]. While marker-based systems are also subject to errors such as soft-tissue artifact and marker placement variability at the ankle, the nature and magnitude of the errors differ between the approaches [23,24]. This distinction reinforces the conclusion that the two systems are not directly interchangeable.

Several methodological factors likely account for the observed error patterns. First, the disparity in sampling frequency (Vicon: 200 Hz; MocapGS: 50 Hz) may be critical. The lower rate of MocapGS could be insufficient to accurately capture rapid distal-segment movements during slow running, potentially introducing aliasing or loss of kinematic detail [25]. Second, although the four-camera setup provided a calibrated volume, it presented challenges for dynamic tasks. Occlusion of the ankle and lower leg during slow running could destabilize the SoG model fitting, an effect particularly detrimental for estimating smaller-magnitude rotations in non-sagittal planes. Beyond measurement constraints, fundamental modeling differences must be considered. Inherent variations in coordinate system definitions and joint center estimation methods between the Plug-in Gait model and the proprietary SoG-based algorithm can lead to systematic offsets in the reported ROM [3,14,26], which further explain the lack of agreement between systems.

### 4.3. Practical Implications for Clinical and Research Applications

Our findings provide concrete guidance for practitioners considering the adoption of MocapGS. This system demonstrates acceptable reliability for monitoring the sagittal-plane range of motion at the hip and knee during gait. This suggests its potential utility in applied settings where tracking gross movement patterns is sufficient, such as in longitudinal rehabilitation monitoring or large-scale biomechanical screening. However, the outputs require careful interpretation for ankle kinematics and for motions in the frontal and transverse planes at all joints, where measurement errors were larger and reliability was often poor.

The RMSE between systems frequently exceeded 5 degrees for many joint-plane combinations, a threshold often referenced for clinically meaningful differences in gait kinematics [27,28]. This underscores that changes smaller than this margin may be indistinguishable from system noise. Consequently, the current MocapGS system is not a substitute for marker-based motion capture in applications demanding high precision, such as clinical decision-making or the detection of subtle gait alterations [29,30].

Building on these performance boundaries, the system’s suitability varies by application: (1) For sports monitoring and large-scale screening, it may be appropriate for identifying gross deviations in lower-limb movement patterns, especially in the sagittal plane, within scalable workflows. (2) In rehabilitation settings, it could support the longitudinal tracking of broad kinematic trends during recovery. (3) For clinical gait laboratories, the evidence does not support its use as a replacement for marker-based systems in diagnostic decision-making or the detection of subtle pathological changes; its role would currently be limited to supplemental screening or monitoring where exact agreement with gold-standard kinematics is not critical [31,32,33].

### 4.4. Future Directions

Future work should aim to improve the robustness and accuracy of SoG-based markerless systems for gait analysis through multi-faceted approaches. This includes optimizing hardware configuration, such as employing more cameras, enhancing the algorithmic core by improving model fitting under occlusion, and exploring advanced data augmentation and balancing strategies, such as generative AI techniques to synthesize training data for challenging motions [34]. Methodological harmonization and standardization should also be promoted [35,36]. Additional validation should include larger and more diverse samples, multiple gait speeds, and clinical populations and examine not only ROM but also time-series kinematics and other relevant parameters [3,36,37,38].

## 5. Conclusions

This study evaluated the reliability and concurrent validity of an SoG-based markerless motion capture system (MocapGS) for estimating bilateral lower-limb joint ROM during walking and slow running, using a marker-based Vicon system as the reference. The MocapGS system demonstrated acceptable test–retest reliability primarily for the sagittal-plane ROM at the hip and knee, whereas reliability and agreement were poor for the non-sagittal-plane ROM and ankle kinematics. Therefore, the current MocapGS system may be suitable for coarse monitoring of large-magnitude sagittal-plane kinematic changes in gait, but it is not yet a substitute for marker-based motion capture in applications requiring precise multi-planar kinematics or detection of subtle gait changes. From a translational standpoint, these findings provide an evidence base for using markerless gait assessment in footwear biomechanics and sports injury prevention scenarios that prioritize scalable screening and longitudinal monitoring over fine-grained clinical decision thresholds.

## Figures and Tables

**Table 1:** ICCs for ROM measured by the Vicon and MocapGS systems during walking.

Joint	Axis	System	Left Lower Limb			Right Lower Limb		
			ICC [95% CI]	F	*p*	ICC [95% CI]	F	*p*
Ankle	X	Vicon	0.44 [−0.03, 0.75]	2.50	0.034	0.69 [0.36, 0.87]	5.63	<0.001
		MocapGS	0.32 [−0.17, 0.68]	1.92	0.095	0.40 [−0.09, 0.72]	2.24	0.053
	Y	Vicon	0.75 [0.44, 0.90]	6.67	<0.001	0.86 [0.67, 0.95]	12.79	<0.001
		MocapGS	0.20 [−0.30, 0.60]	1.48	0.216	−0.20 [−0.63, 0.30]	0.68	0.786
	Z	Vicon	0.59 [0.17, 0.83]	3.72	0.005	0.96 [0.90, 0.99]	50.79	<0.001
		MocapGS	0.26 [−0.25, 0.65]	1.66	0.153	0.43 [0.01, 0.73]	2.84	0.019
Knee	X	Vicon	0.39 [−0.03, 0.71]	2.68	0.025	0.74 [0.43, 0.90]	6.41	<0.001
		MocapGS	0.50 [0.05, 0.78]	2.91	0.017	0.28 [−0.21, 0.66]	1.77	0.125
	Y	Vicon	0.97 [0.92, 0.99]	62.62	<0.001	0.75 [0.46, 0.90]	7.52	<0.001
		MocapGS	0.49 [0.06, 0.77]	2.93	0.016	−0.05 [−0.53, 0.44]	0.92	0.568
	Z	Vicon	0.85 [0.65, 0.94]	13.65	0.000	0.89 [0.72, 0.96]	18.10	<0.001
		MocapGS	0.44 [−0.02, 0.75]	2.55	0.031	0.74 [0.44, 0.89]	6.86	<0.001
Hip	X	Vicon	0.91 [0.76, 0.97]	26.54	<0.001	0.96 [0.90, 0.99]	53.78	<0.001
		MocapGS	0.76 [0.47, 0.90]	7.14	<0.001	0.56 [0.16, 0.80]	3.79	0.004
	Y	Vicon	0.87 [0.69, 0.95]	13.92	<0.001	0.79 [0.54, 0.92]	8.85	<0.001
		MocapGS	0.07 [−0.44, 0.52]	1.14	0.397	−0.10 [−0.50, 0.46]	0.98	0.515
	Z	Vicon	0.77 [0.48, 0.91]	7.31	<0.001	0.95 [0.87, 0.98]	40.64	<0.001
		MocapGS	0.29 [−0.22, 0.67]	1.78	0.122	−0.01 [−0.50, 0.46]	0.99	0.521

Note: ICC is the intraclass correlation coefficient. 95% CI is 95% confidence interval.

**Table 2:** The SEM and SEM% for ROM measured by the Vicon and MocapGS systems during walking.

Joint	Axis	System	Left Lower Limb			Right Lower Limb		
			ROM	SEM	SEM%	ROM	SEM	SEM%
Ankle	X	Vicon	29.50 ± 2.87	2.14	7.27	27.50 ± 3.47	1.93	7.00
		MocapGS	41.42 ± 4.45	3.67	8.86	42.81 ± 5.2	4.04	9.44
	Y	Vicon	19.66 ± 6.84	3.45	17.53	17.07 ± 2.98	1.12	6.53
		MocapGS	9.45 ± 3.30	2.96	31.30	8.60 ± 2.37	2.60	30.22
	Z	Vicon	19.01 ± 4.87	3.12	16.42	18.85 ± 5.81	1.13	6.01
		MocapGS	0.00 ± 0.01	0.01	35.77	0.02 ± 0.01	0.00	23.41
Knee	X	Vicon	37.09 ± 5.45	4.27	11.50	38.09 ± 3.26	1.66	4.36
		MocapGS	59.04 ± 5.36	3.80	6.43	62.05 ± 5.58	4.72	7.61
	Y	Vicon	10.66 ± 4.29	0.74	6.97	9.77 ± 1.26	0.63	6.45
		MocapGS	12.72 ± 5.02	3.60	28.28	8.13 ± 2.55	2.61	32.07
	Z	Vicon	15.55 ± 3.00	1.15	7.39	16.49 ± 3.97	1.33	8.08
		MocapGS	12.77 ± 5.50	4.11	32.21	13.84 ± 4.65	2.37	17.15
Hip	X	Vicon	50.95 ± 4.89	1.61	3.16	50.64 ± 5.04	0.64	1.27
		MocapGS	50.68 ± 6.21	3.07	6.05	51.26 ± 5.07	3.38	6.60
	Y	Vicon	14.88 ± 2.04	1.53	10.31	12.81 ± 1.67	0.57	4.48
		MocapGS	9.68 ± 2.19	2.11	21.83	13.33 ± 2.96	3.11	23.31
	Z	Vicon	11.91 ± 2.20	1.45	12.17	14.14 ± 4.81	0.90	6.34
		MocapGS	9.49 ± 3.90	3.28	34.60	13.71 ± 5.21	5.23	38.17

Note: ROM is the range of motion. SEM is the standard error of measurement. SEM% is the SEM percentage.

**Table 3:** The MDC and MDC% for ROM measured by the Vicon and MocapGS systems during walking.

Joint	Axis	System	Left Lower Limb			Right Lower Limb		
			ROM	MDC	MDC%	ROM	MDC	MDC%
Ankle	X	Vicon	29.50 ± 2.87	5.94	20.15	27.50 ± 3.47	5.34	19.41
		MocapGS	41.42 ± 4.45	10.17	24.56	42.81 ± 5.2	11.20	26.17
	Y	Vicon	19.66 ± 6.84	9.55	48.59	17.07 ± 2.98	3.09	18.11
		MocapGS	9.45 ± 3.30	8.20	86.77	8.60 ± 2.37	7.21	83.77
	Z	Vicon	19.01 ± 4.87	8.65	45.50	18.85 ± 5.81	3.14	16.65
		MocapGS	0.00 ± 0.01	0.02	99.14	0.02 ± 0.01	0.01	64.88
Knee	X	Vicon	37.09 ± 5.45	11.83	31.89	38.09 ± 3.26	4.60	12.08
		MocapGS	59.04 ± 5.36	10.53	17.83	62.05 ± 5.58	13.08	21.09
	Y	Vicon	10.66 ± 4.29	2.06	19.31	9.77 ± 1.26	1.75	17.89
		MocapGS	12.72 ± 5.02	9.97	78.39	8.13 ± 2.55	7.23	88.89
	Z	Vicon	15.55 ± 3.00	3.19	20.49	16.49 ± 3.97	3.70	22.40
		MocapGS	12.77 ± 5.50	11.40	89.28	13.84 ± 4.65	6.58	47.55
Hip	X	Vicon	50.95 ± 4.89	4.46	8.75	50.64 ± 5.04	1.78	3.52
		MocapGS	50.68 ± 6.21	8.50	16.76	51.26 ± 5.07	9.37	18.29
	Y	Vicon	14.88 ± 2.04	4.25	28.57	12.81 ± 1.67	1.59	12.41
		MocapGS	9.68 ± 2.19	5.86	60.52	13.33 ± 2.96	8.61	64.61
	Z	Vicon	11.91 ± 2.20	4.02	33.75	14.14 ± 4.81	2.48	17.56
		MocapGS	9.49 ± 3.90	9.09	95.91	13.71 ± 5.21	14.50	105.79

Note: ROM is the range of motion. MDC is the minimal detectable change. MDC% is the MDC percentage.

**Table 4:** Comparison of ROM measured by the Vicon and MocapGS systems during walking (degrees, M ± SD).

Joint	Axis	Left Lower Limb				Right Lower Limb			
		t/Z	*p*	Effect Size	RMSE (°)	t/Z	*p*	Effect Size	RMSE (°)
Ankle	X	13.7	<0.001	0.84	13.06	13.22	<0.001	0.83	16.77
	Y	8.64	<0.001	0.68	12.37	−5.18	<0.001	0.74	9.26
	Z	23.40	<0.001	0.94	19.59	−5.23	<0.001	0.76	19.69
Knee	X	15.99	<0.001	0.88	23.41	−5.23	<0.001	0.76	24.10
	Y	1.91	0.064	0.09	6.70	3.41	0.002	0.25	3.27
	Z	3.01	0.005	0.21	6.12	2.88	0.007	0.20	6.06
Hip	X	−0.77	0.451	0.02	7.60	0.62	0.541	0.01	5.96
	Y	12.26	<0.001	0.81	5.77	0.88	0.383	0.02	3.49
	Z	3.13	0.004	0.22	5.21	−0.82	0.421	0.02	7.03

Note: RMSE is the root mean square error.

**Table 5:** Agreement analysis of ROM between Vicon and MocapGS during walking.

Joint	Axis	CCC [95% CI]	*p*	Mean Difference (Bias) [95% CI]	Standard Deviation of Differences	Upper 95% LOA [95% CI]	Lower 95% LOA [95% CI]
Left lower limb							
Ankle	X	0.00 [−0.05, 0.05]	0.896	−11.99 [−13.73, −10.24]	5.25	−1.69 [−24.03, −20.54]	−22.28 [−3.44, 0.05],
	Y	0.05 [−0.03, 0.14]	0.333	10.21 [7.81, 12.60]	7.09	24.09 [19.94, 28.24]	−3.68 [−7.83, 0.47]
	Z	−0.00 [−0.00, 0.00]	0.149	18.99 [17.35, 20.64]	4.87	28.54 [25.69, 31.39]	9.45 [6.60, 12.30]
Knee	X	−0.02 [−0.05, 0.01]	0.357	−21.96 [−24.74, −19.17]	8.24	−5.81 [−10.63, −0.98]	−38.10 [−42.93, −33.28]
	Y	0.04 [−0.25, 0.34]	0.815	−2.06 [−4.25, 0.13]	6.46	10.61 [10.61, 14.38]	−14.73 [−18.51, −14.73]
	Z	0.18 [−0.02, 0.40]	0.126	2.78 [0.91, 4.65]	5.53	13.63 [10.39, 16.87]	−8.07 [−11.31, −4.82]
Hip	X	0.05 [−0.18, 0.30]	0.784	0.27 [−2.34, 2.87]	7.70	15.36 [−0.49, 31.21]	−14.83 [−30.68, 1.02]
	Y	0.07 [−0.01, 0.15]	0.104	5.20 [4.34, 6.06]	2.55	10.19 [8.71, 11.67]	0.21 [−1.27, 1.69]
	Z	−0.07 [−0.30, 0.19]	0.545	2.44 [0.89, 3.86]	4.67	11.59 [9.09, 13.78]	−6.71 [−10.05, −3.46]
Right lower limb							
Ankle	X	−0.03 [−0.09, 0.00]	0.131	−15.31 [−17.66, −12.96]	6.95	−1.69 [−4.58, 1.21]	−28.93 [−31.82, −26.04]
	Y	0.00 [−0.07, 0.06]	0.983	8.47 [7.18, 9.76]	3.80	15.92 [14.34, 17.51]	1.02 [−0.57, 2.60]
	Z	0.00 [−0.00, 0.00]	0.407	18.83 [16.87, 20.80]	5.81	30.22 [27.80, 32.64]	7.45 [5.03, 9.87]
Knee	X	−0.01 [−0.03, 0.01]	0.352	−23.96 [−26.29, −21.62]	6.90	−10.43 [−13.30, −7.56]	−37.49 [−40.36, −34.61]
	Y	−0.01 [−0.19, 0.14]	0.893	1.63 [0.66, 2.61]	2.87	7.26 [6.07, 8.46]	−4.00 [−5.19, −2.80]
	Z	0.15 [−0.09, 0.37]	0.277	2.65 [0.79, 4.52]	5.52	13.48 [11.18, 15.78]	−8.17 [−10.47, −5.87]
Hip	X	0.29 [−0.05, 0.56]	0.084	−0.62 [−2.65, 1.42]	6.01	11.17 [8.67, 13.68]	−12.41 [−14.91, −9.90]
	Y	−0.06 [−0.36, 0.23]	0.694	−0.52 [−1.70, 0.67]	3.50	6.34 [4.89, 7.80]	−7.37 [−8.83, −5.92]
	Z	−0.01 [−0.35, 0.37]	0.977	0.43 [−1.98, 2.84]	7.11	14.37 [11.41, 17.33]	−13.51 [−16.47, −10.55]

Note: CCC is the concordance correlation coefficient. 95% LOA is the 95% limits of agreement. 95% CI is the 95% confidence interval.

**Table 6:** The ICCs for ROM measured by the Vicon and MocapGS systems during slow running.

Joint	Axis	System	Left Lower Limb				Right Lower Limb			
			ROM	ICC [95% CI]	F	*p*	ROM	ICC [95% CI]	F	*p*
Ankle	X	Vicon	48.11 ± 5.87	0.86 [0.67, 0.95]	13.16	<0.001	49.61 ± 7.37	0.86 [0.67, 0.95]	12.81	<0.001
		MocaGS	55.09 ± 5.53	0.75 [0.46, 0.90]	7.12	<0.001	53.11 ± 5.96	0.27 [−0.16, 0.63]	1.81	0.115
	Y	Vicon	24.24 ± 5.90	0.92 [0.80, 0.971]	27.77	<0.001	24.46 ± 3.07	0.62 [−0.16, 0.63]	4.09	0.003
		MocapGS	7.46 ± 2.80	0.44 [0.02, 0.74]	2.89	0.018	7.71 ± 2.81	0.36 [−0.06, 0.69]	2.29	0.048
	Z	Vicon	20.15 ± 5.12	0.65 [0.22, 0.86]	6.03	<0.001	15.83 ± 1.73	0.16 [−0.35, 0.58]	1.36	0.268
		MocapGS	0.02 ± 0.00	−0.18 [−0.62, 0.32]	0.71	0.759	0.02 ± 0.01	−0.23 [0.67, 0.28]	0.65	0.809
Knee	X	Vicon	27.10 ± 1.55	−0.81 [0.56, 0.41]	0.86	0.621	30.87 ± 4.27	0.81 [0.56, 0.92]	9.94	<0.001
		MocapGS	63.44 ± 6.57	0.47 [0.05, 0.76]	2.99	0.015	67.24 ± 9.63	0.57 [0.13, 0.81]	3.46	0.007
	Y	Vicon	14.37 ± 1.89	0.70 [0.35, 0.88]	6.41	<0.001	12.70 ± 4.94	0.96 [0.81, 0.99]	73.41	<0.001
		MocapGS	9.43 ± 3.65	0.29 [−0.15, 0.65]	1.90	0.099	7.95 ± 2.11	0.23 [−0.20, 0.61]	1.66	0.153
	Z	Vicon	19.22 ± 1.04	0.89 [0.73, 0.96]	16.50	<0.001	19.22 ± 1.04	0.12 [−0.38, 0.55]	1.27	0.316
		MocapGS	11.77 ± 5.57	0.31 [−0.11, 0.66]	2.06	0.074	14.63 ± 5.88	0.18 [0.13, 0.81]	0.95	0.418
Hip	X	Vicon	42.08 ± 7.42	0.90 [0.74, 0.96]	17.33	<0.001	40.99 ± 6.08	0.96 [0.87, 0.98]	52.02	<0.001
		MocapGS	47.69 ± 9.27	0.85 [0.64, 0.94]	12.29	<0.001	46.16 ± 11.55	0.84 [0.62, 0.94]	12.46	<0.001
	Y	Vicon	12.35 ± 2.95	0.83 [0.61, 0.93]	10.73	<0.001	12.48 ± 2.85	0.84 [0.61, 0.94]	12.34	<0.001
		MocapGS	6.72 ± 2.38	−0.11 [−0.48, 0.34]	0.79	0.689	13.79 ± 4.23	0.32 [−0.19, 0.68]	1.88	0.102
	Z	Vicon	11.67 ± 3.55	0.94 [0.85, 0.98]	31.65	<0.001	16.92 ± 4.53	0.98 [0.95, 0.99]	102.79	<0.001
		MocapGS	10.40 ± 4.11	−0.08 [−0.56, 0.41]	0.86	0.620	13.21 ± 4.73	0.30 [−0.15, 0.66]	1.92	0.095

Note: ROM is the range of motion. ICC is the intraclass correlation coefficient. 95% CI is the 95% confidence interval.

**Table 7:** The SEM, SEM%, MDC, and MDC% for ROM measured by the Vicon and MocapGS systems during slow running.

Joint	Axis	System	Left Lower Limb				Right Lower Limb			
			SEM	SEM%	MDC	MDC%	SEM	SEM%	MDC	MDC%
Ankle	X	Vicon	2.18	4.53	6.04	12.56	2.74	5.52	7.59	15.30
		MocapGS	2.77	5.02	7.66	13.91	5.11	9.62	14.17	26.68
	Y	Vicon	1.65	6.79	4.56	18.83	1.90	7.75	5.26	21.48
		MocapGS	2.09	28.07	5.80	77.80	2.25	29.17	6.24	80.85
	Z	Vicon	3.03	15.06	8.41	41.74	1.59	10.04	4.41	27.84
		MocapGS	0.01	34.68	0.01	96.14	0.01	42.36	0.02	117.42
Knee	X	Vicon	2.08	7.69	5.78	21.32	1.88	6.09	5.21	16.89
		MocapGS	4.78	7.54	13.25	20.89	6.35	9.45	17.60	26.18
	Y	Vicon	1.04	7.24	2.88	20.06	0.99	7.78	2.74	21.56
		MocapGS	3.07	32.59	8.52	90.33	1.85	23.27	5.13	64.49
	Z	Vicon	0.78	4.04	2.16	11.20	0.98	5.08	2.71	14.08
		MocapGS	4.63	39.31	12.82	108.97	5.33	36.47	14.79	101.08
Hip	X	Vicon	0.50	1.19	1.39	3.29	0.91	2.21	2.51	6.13
		MocapGS	3.64	7.62	10.08	21.13	4.62	10.01	12.80	27.73
	Y	Vicon	0.78	6.30	2.16	17.46	2.01	16.07	5.56	44.56
		MocapGS	2.51	37.33	6.95	103.46	3.50	25.37	9.70	70.32
	Z	Vicon	0.57	4.92	1.59	13.64	0.14	0.83	0.39	2.29
		MocapGS	4.27	41.08	11.84	113.86	3.94	29.87	10.93	82.79

Note: SEM is the standard error of measurement. SEM% is the SEM percentage. MDC is the minimal detectable change. MDC% is the MDC percentage. The mean ROM values used to calculate the SEM% and MDC% are provided in Table 6.

**Table 8:** Comparison of ROM and RMSE measured by the Vicon and MocapGS systems during slow running.

Joint	Axis	Left Lower Limb				Right Lower Limb			
		t/Z	*p*	Effect Size	RMSE (°)	t/Z	*p*	Effect Size	RMSE (°)
Ankle	X	5.66	<0.001	0.48	10.10	2.20	0.035	0.12	10.05
	Y	−5.23	<0.001	0.76	18.02	23.01	<0.001	0.94	17.24
	Z	−5.23	<0.001	0.76	20.76	−5.23	<0.001	0.76	15.90
Knee	X	31.12	<0.001	0.97	36.99	20.42	<0.001	0.92	37.86
	Y	6.55	<0.001	0.55	6.67	5.35	<0.001	0.45	7.07
	Z	7.44	<0.001	0.61	9.57	4.58	<0.001	0.37	7.51
Hip	X	3.33	0.002	0.24	11.44	2.77	0.009	0.18	12.20
	Y	8.86	<0.001	0.69	6.78	1.59	0.122	0.07	5.08
	Z	1.47	0.150	0.06	5.30	3.29	0.002	0.24	7.65

Note: RMSE is the root mean square error.

**Table 9:** Agreement statistics for ROM between Vicon and MocapGS during slow running.

Joint	Axis	CCC [95% CI]	*p*	Mean Difference (Bias) [95% CI]	Standard Deviation of Differences	Upper 95% LOA [95% CI]	Lower 95% LOA [95% CI]
Left lower limb							
Ankle	X	0.09 [−0.08, 0.25]	0.365	−6.98 [−9.48, −4.47]	7.40	7.53 [3.21, 11.85]	−21.49 [−25.81, −17.17]
	Y	−0.01 [−0.04, 0.04]	0.770	16.78 [14.53, 19.04]	6.66	29.83 [25.94, 33.71]	3.74 [0.15, 7.62]
	Z	−0.00 [−0.00, 0.00]	0.791	20.14 [18.40, 21.87]	5.12	30.16 [27.18, 33.15]	10.11 [7.12, 13.09]
Knee	X	−0.00 [−0.01, 0.00]	0.306	−36.33 [−38.71, −33.97]	7.01	−22.61 [−26.69, −18.52]	−50.07 [−54.16, −45.98]
	Y	−0.09 [−0.22, 0.03]	0.129	4.94 [3.41, 6.47]	4.52	13.81 [11.17, 16.45]	−3.93 [−6.57, −1.28]
	Z	−0.00 [−0.13, 0.11]	0.980	7.50 [5.45, 9.54]	6.04	19.34 [15.81, 22.87]	−4.35 [−7.88, −0.82]
Hip	X	0.22 [−0.07, 0.48]	0.097	−5.61 [−9.03, −2.19]	10.11	14.21 [8.30, 20.11]	−25.43 [−31.33, −19.53]
	Y	−0.00 [−0.09, 0.08]	0.942	5.64 [4.34, 6.93]	3.82	13.12 [10.89, 15.35]	−1.85 [−4.08, 0.38]
	Z	0.07 [−0.31, 0.44]	0.646	1.28 [−0.49, 3.04]	5.22	11.50 [8.46, 14.55]	−8.95 [−11.99, −5.90]
Right lower limb							
Ankle	X	−0.01 [−0.28, 0.25]	0.926	−3.50 [−6.73, −0.27]	9.55	15.22 [9.84, 20.60]	−22.22 [−27.60, −16.83]
	Y	0.00 [−0.02, 0.03]	0.851	16.19 [14.48, 17.90]	5.05	26.09 [23.24, 28.93]	6.30 [3.45, 9.14]
	Z	−0.00 [−0.00, 0.00]	0.553	15.81 [15.22, 16.40]	1.73	19.21 [18.23, 20.18]	12.41 [11.44, 13.39]
Knee	X	−0.00 [−0.02, 0.01]	0.826	−36.37 [−39.99, −32.75]	10.69	−15.42 [−21.45, −9.40]	−57.31 [−63.33, −51.29]
	Y	0.01 [−0.12, 0.15]	0.886	4.75 [2.95, 6.55]	5.32	15.17 [12.17, 18.17]	−5.68 [−8.68, −2.68]
	Z	−0.01 [−0.10, 0.07]	0.773	4.60 [2.56, 6.64]	6.02	16.40 [13.01, 19.79]	−7.21 [−10.60, −3.82]
Hip	X	0.23 [−0.03, 0.46]	0.063	−5.17 [−8.96, −1.38]	11.20	16.80 [10.48, 23.11]	−27.14 [−33.45, −20.82]
	Y	0.05 [−0.20, 0.32]	0.759	−1.32 [−3.00, 0.36]	4.98	8.45 [5.64, 11.25]	−11.08 [−13.88, −8.27]
	Z	−0.05 [−0.32, 0.18]	0.684	3.71 [1.42, 6.00]	6.78	17.00 [13.18, 20.82]	−9.57 [−13.39, −5.75]

Note: CCC is the concordance correlation coefficient. 95% LOA is the 95% limits of agreement. 95% CI is the 95% confidence interval.

## Data Availability

The data are available on request from the corresponding author.

## Abbreviations

The following abbreviations are used in this manuscript:

SoG	sums-of-Gaussians
ROM	range of motion
ICC	intraclass correlation coefficient
SEM	standard error of measurement
SEM%	SEM percentage
MDC	minimal detectable change
MDC%	MDC percentage
RMSE	root mean square error
SD	standard deviation
CCC	concordance correlation coefficient
LOA	limits of agreement
95% CI	95% confidence interval
MD	mean difference
Ankle_L	left ankle joint
Ankle_R	right ankle joint
Knee_L	left knee joint
Knee_R	right knee joint
Hip_L	left hip joint
Hip_R	right hip joint
Ankle_L_X	left ankle joint in the sagittal plane
Ankle_R_X	right ankle joint in the sagittal plane
Ankle_L_Y	left ankle joint in the frontal plane
Ankle_R_Y	right ankle joint in the frontal plane
Ankle_L_Z	left ankle joint in the transverse plane
Ankle_R_Z	right ankle joint in the transverse plane
Knee_L_X	left knee joint in the sagittal plane
Knee_R_X	right knee joint in the sagittal plane
Knee_L_Y	left knee joint in the frontal plane
Knee_R_Y	right knee joint in the frontal plane
Knee_L_Z	left knee joint in the transverse plane
Knee_R_Z	right knee joint in the transverse plane
Hip_L_X	left hip joint in the sagittal plane
Hip_R_X	right knee joint in the sagittal plane
Hip_L_Y	left hip joint in the frontal plane
Hip_R_Y	right hip joint in the frontal plane
Hip_L_Z	left hip joint in the transverse plane
Hip_R_Z	right hip joint in the transverse plane
